# Supplementary material for: IMMUNOTAR - Integrative prioritization of cell surface targets for cancer immunotherapy
Source: bioRxiv. 2024 Jun 6:2024.06.04.597422. Preprint. [Version 1] doi: 10.1101/2024.06.04.597422 (PMC11185603; doi:10.1101/2024.06.04.597422)
Supplement: Supplement 3 [file media-3.pdf]

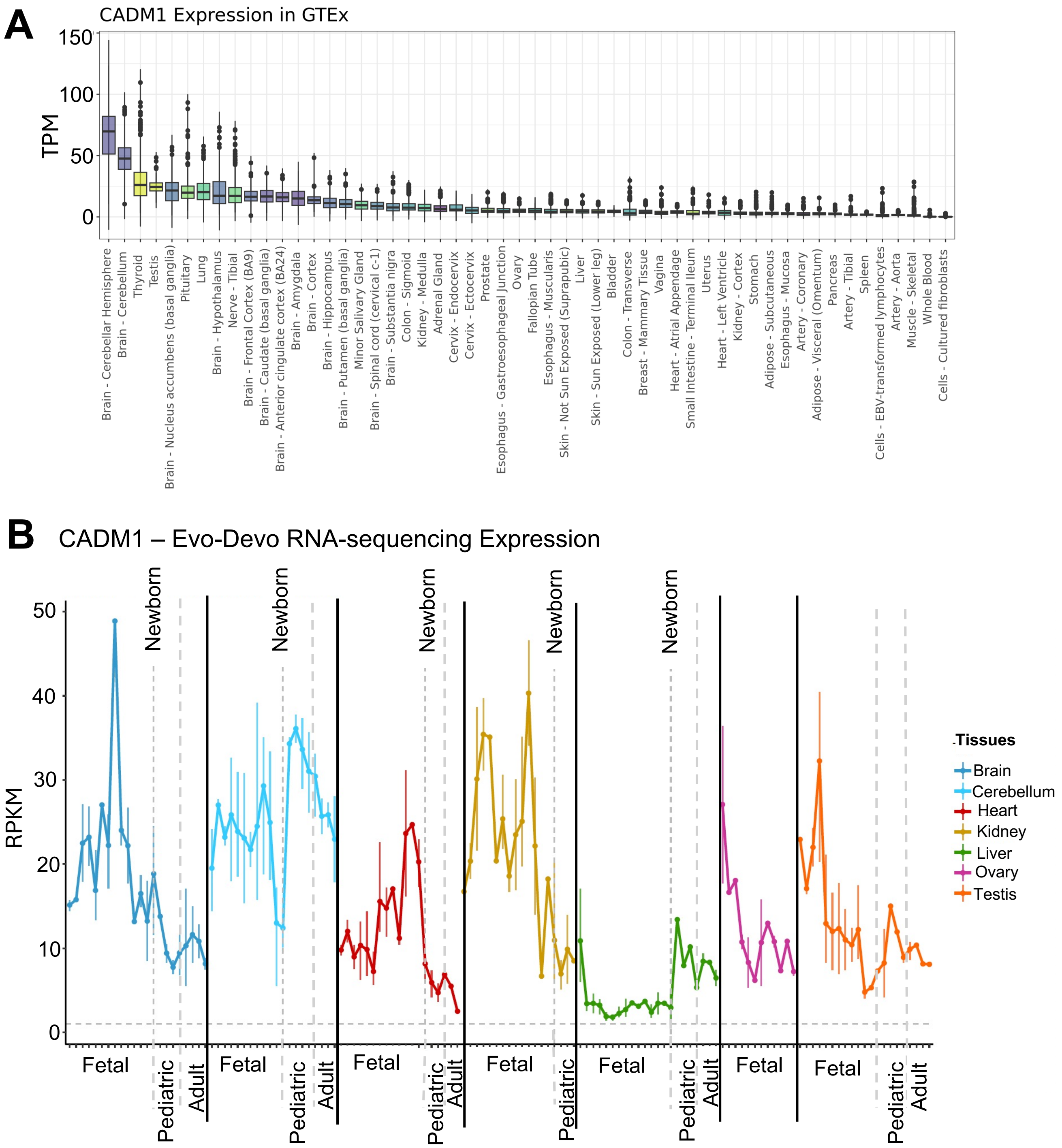

Supplemental Fig. S1: *CADM1* expression in normal tissues databases GTEx and Evo-Devo. **A)** Normal tissue expression per GTEx RNA-sequencing database. **B)** Normal tissue expression per Evo-Devo RNA-sequencing database.

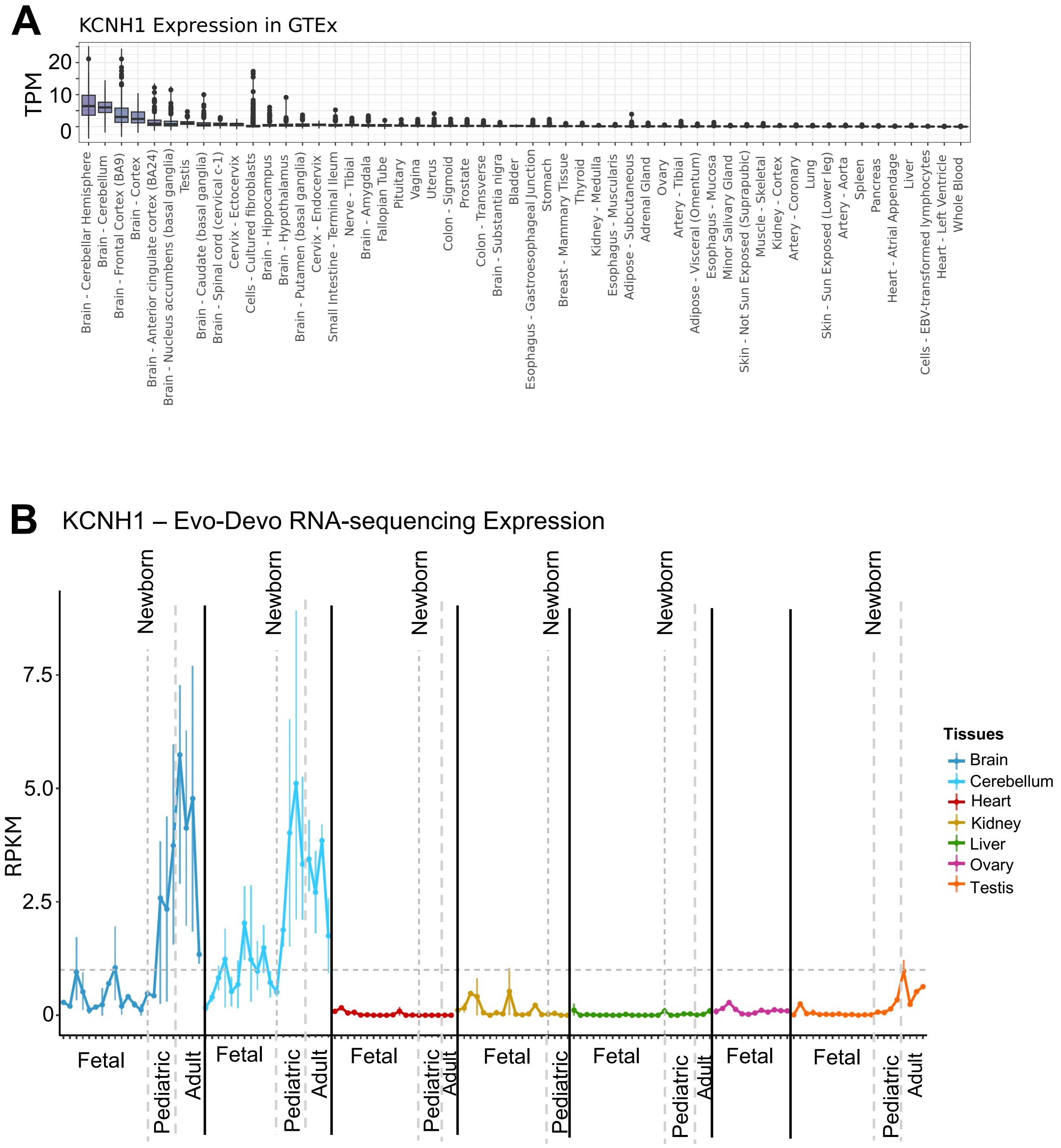

Supplementary Fig. S2: ***KCNH1* expression in normal tissues databases GTEx and Evo-Devo**. **A)** Normal tissue expression per GTEx RNA-sequencing database. **B)** Normal tissue expression per Evo-Devo RNA-sequencing database.
